# Dentistry at the Paris Exposition

**Published:** 1868-02

**Authors:** 


### DENTISTRY AT THE PARIS EXPOSITION.

A QUARTER of a century ago, we became acquainted with the experiments of a dentist, in the effort to produce artificial teeth in a continuous gum, of a natural color and appearance, and which would not warp or become fissured in use. We sometimes visited his house and laboratory — almost daily — making minute inquiries and examinations of the materials, nature, and qualities, and progress of the work. During the last year or two we have not spoken to him, although living within a few minutes' walk of his residence, so neglectful are old friends of each other in this whirling, whirlpool city, unless stern business, or sterner illness, brings them together; but we had heard of his sending specimens of his workmanship to the great Paris Exposition, and had been wondering in our own minds why we had not seen his name, with the grand cross of the legion of a dozen honors, we unexpectedly learned that the managers of the Exposition were not willing to place the efforts of genius, the work of professional men, with that of artificers. These labors continued for several years, through many discouragements and partial failures; but, with a perseverance which belongs to men of true talent and high ability, he was able, at length,. to arrive at a success which we felt confident would eventually be rewarded with a due appreciation, not only by the public at large, but by the dental profession of both hemispheres, which has been done. The improvements of dentists, physicians, and surgeons were diffferently classified, and the awards of merit granted to them were designated by official reports from the imperial commission, while the awards of the artisans and manufacturers were represented simply by medals.

### "INCOMPARABLY BEAUTIFUL."

The jury on dentistry at the Champs de Mars have reported that "The specimens of 'Continuous gum sets of teeth, upon platinum plate,' by John Allen & Son, of New-York, are incomparably the most beautiful pieces exhibited." This report has been corroborated by various newspaper correspondents writing from Paris.

"BEYOND ALL COMPETITION."

The New-York *Times*, of May 14th, 1867, has an article from its correspondent, saying of American dentistry, "It is beyond all competition. The display of artificial teeth, the perfect imitation of nature, in gums, and palate, or roof, are wonderful. But the French are equally great in eyes and ears."

"NOTHING LIKE THEM."

A Philadelphia correspondent, writing from Paris, April 15th, 1867, says: "The case of teeth by J. Allen & Son attracts much attention. It is very much admired. Nothing like it seems to be known here; and people ask all sorts of questions regarding them. They are far in advance of the times here."

"SURPASSES EVERY THING."

A New-York correspondent writes: "Paris, June 12th, 1867. I have conversed with many persons here on the subject of dentistry on exhibition, and they all agree that the Artificial Dentures of J. Allen & Son surpasses any and every thing of the kind here represented."

A Parisian dentist writes to a friend in New-York, July 13th, 1867: "I embrace this opportunity of testifying to the excellence and superiority, above all others, of the dental work here of Allen & Son." Another Parisian dentist writes: "You may feel sure there is nothing in the whole exhibition of dentistry which can in any way approach the perfection and beauty of the dental work of Allen & Son."

The above official report from the old world is reëchoed by the American Institute at their great Fair, held in New-York City, during October, 1867, when another medal was awarded J. Allen & Son, together with the following report from the Judges of Dentistry: "Case No. 508, Mounted Artificial Teeth on Platinum Base, by J. Allen & Son, No. 22 Bond street, New-York City,

THE BEST ON EXHIBITION.

Their merits are strength, durability, cleanliness, and adaptation to every conceivable physiognomical requirement of the teeth, and color of the gums."

